# Trends in incidence rate, health care use, and costs due to rib fractures in the Netherlands

**DOI:** 10.1007/s00068-021-01662-8

**Published:** 2021-04-12

**Authors:** Jonne T. H. Prins, Mathieu M. E. Wijffels, Sophie M. Wooldrik, Martien J. M. Panneman, Michael H. J. Verhofstad, Esther M. M. Van Lieshout

**Affiliations:** 1 Trauma Research Unit, Department of Surgery, Erasmus MC, University Medical Center Rotterdam, P.O. Box 2040, 3000 CA Rotterdam, The Netherlands; 2 Consumer Safety Institute, Amsterdam, The Netherlands

**Keywords:** Rib fractures, Epidemiology, Incidence rate, Hospital length of stay, Health care costs, Health care consumption

## Abstract

**Purpose:**

This study aimed to examine population-based trends in the incidence rate, health care consumption, and work absence with associated costs in patients with rib fractures.

**Methods:**

A retrospective nationwide epidemiologic study was performed with data from patients with one or more rib fractures presented or admitted to a hospital in the Netherlands between January 1, 2015 and December 31, 2018 and have been registered in the Dutch Injury Surveillance System (DISS) or the Hospital Discharge Registry (HDR). Incidence rates were calculated using data from Statistics Netherlands. The associated direct health care costs, costs for lost productivity, and years lived with disability (YLD) were calculated using data from a questionnaire.

**Results:**

In the 4-year study period, a total of 32,124 patients were registered of which 19,885 (61.9%) required hospitalization with a mean duration of 7.7 days. The incidence rate for the total cohort was 47.1 per 100,000 person years and increased with age. The mean associated direct health care costs were €6785 per patient and showed a sharp increase after the age of 75 years. The mean duration of work absence was 44.2 days with associated mean indirect costs for lost productivity of €22,886 per patient. The mean YLD was 0.35 years and decreased with age.

**Conclusion:**

Rib fractures are common and associated with lengthy HLOS and work absenteeism as well as high direct and indirect costs which appear to be similar between patients with one or multiple rib fractures and mostly affected by admitted patients and age.

## Introduction

Thoracic wall injury after blunt chest trauma is common and rib fractures are diagnosed in 10% of patients after trauma and 30% of patients after blunt thoracic trauma [1–3]. In younger patients, most rib fractures are caused by a high-energy trauma (HET), whereas more than half of the patients aged 65 years or older sustain rib fractures following low-energy trauma (LET) [4, 5]. Rib fractures can occur as single or multiple simple rib fractures or as a flail segment in which three or more rib fractures are fractured in two or more places [6]. Patient and injury characteristics influence the outcome after rib fractures. Increased age, increased number of rib fractures, and presence of concomitant thoracic injuries are associated with poorer outcome including higher pneumonia risk, increased hospital and Intensive Care Unit length of stay (HLOS and ICLOS, respectively), and increased mortality [5, 7–12]. While negatively affecting in-hospital outcome, rib fractures are also associated with long-term disability, chronic pain, and reduction of quality of life [13–16]. At 2 years post-injury, almost one-third of patients has not yet returned to their pre-injury work level [17]. While the prevalence of rib fractures is known, the incidence rate of rib fractures has only been studied in hospitalized patients or the elderly [18–20]. Although the disabling effect of rib fractures on short- and long-term outcomes is indisputable, the economic effect on health care use, work absence, and associated detailed evaluations of direct and indirect costs has hardly been studied. Insight into the occurrence and economic impact of rib fractures can both assist in daily allocation of health care services and provide a projection for the future.

Therefore, the aim of this nationwide study was to examine population-based trends in the incidence rate of rib fractures for a 4-year period (2015–2018) and to give a detailed overview of the health care consumption and work absence with associated costs in these patients.

## Materials and methods

### Data sources

For this retrospective nationwide epidemiologic study, data from all patients with rib fractures, delineated on chest computed tomography (CT) scan or radiograph, presented to a Dutch hospital between January 1, 2015 and December 31, 2018 were collected. Data were obtained from two different databases: the Dutch Injury Surveillance System (DISS) and the Hospital Discharge Registry (HDR) [21, 22]. “Veiligheid NL” collects this information directly from the Emergency Department (ED) of 14 Dutch hospitals, representing 12% of all injury-related ED visits in the Netherlands. These hospitals are a combination of level 1–3 Trauma Centers, both academic and regional, and geographically distributed across the country with a population that is representative of the Dutch population regarding age and gender. To generate national estimates of injury-related ED visits in the Netherlands, an extrapolation factor was calculated in which the injury-related ED visits registered in the participating hospitals is multiplied by the ratio of nationwide ED visits and DISS ED visits [23]. The DISS database also contains data about health care costs, costs for lost productivity, and quality of life. In the DISS database, the distinction is made between non-admitted patients and admitted patients. Admitted patients are patients who presented to an ED and were immediately admitted to the hospital. Non-admitted patients did not require hospital admission. As all hospitals in the Netherlands are obliged to register data of admitted patients, the data of the HDR have national coverage. The HDR registers all diagnoses, medical procedures, and length of stay in admitted patients. Since the DISS database does not contain patients who are admitted through referral from the outpatient clinic or another hospital and the HDR counts patients who have been admitted more than once (*e.g*., for removal of surgical implants) as a new patient for every single admission, a correction factor has been used.

The DISS database and HDR do not distinguish patients with rib fractures as primary diagnosis or secondary diagnosis. For each outcome measure, the differences in severity of chest wall injury (one or multiple rib fractures, or a flail chest (for HLOS, extracted from the HDR)) and in admitted and non-admitted patients were analyzed. The effect over time was evaluated. Also, the average of the 4 years was determined for each outcome measure. The study was exempted by the local Medical Research Ethics Committee (No. MEC-2020–0179).

### Incidence rate

The primary outcome measure was the age- and gender standardized incidence rate of rib fractures provided by the DISS database. To calculate incidence rates, the study population was divided into age groups (0–24 years, 25–34 years, 35–44 years, etc.) using “direct standardization” as described before [24]. Because a low number of registered patients were younger than 25 years, patients aged 0–24 years were merged into one group. The age-specific incidence rates per 100,000 person years in patients with one or multiple rib fractures were calculated based upon the Dutch mid-year standard population. Mid-year population sizes for all age groups were obtained from Statistics Netherlands [25].

### Hospital length of stay

Data on HLOS were obtained from the HDR database which include the number of patients and the mean hospital length of stay including standard deviation. Mean and cumulative HLOS were calculated for 10-year age groups in patients with either one or multiple rib fractures. To calculate the cumulative HLOS, the number of patients was multiplied with the mean HLOS per patient per age group.

### Costs for health care use

A random sample of patients registered in the DISS database received a follow-up questionnaire on used health care, quality of life (EuroQoL-5D, EQ-5D), and work absence during the first 24 months after trauma [26, 27].

Estimated direct costs due to health care use included the HLOS, ambulance care, general practitioner (GP) visits, in-hospital care, home care, rehabilitation and nursing home care, and physical therapy. Mean and cumulative health care costs were calculated for different age groups, analogous to incidence rate, in patients with either one or multiple rib fractures. Cumulative costs were calculated by multiplying the number of patients by the average medical costs per patient.

### Costs for lost productivity

Costs for lost productivity were divided in duration of work absence and work absence costs. These outcome measures were calculated with the results of the same questionnaire mentioned above which also contained questions on work absenteeism. Work absence costs were defined as costs associated with production loss and replacement due to illness, disability, and premature death. A model to calculate costs due to lost productivity has been developed by VeiligheidNL (“The Dutch Burden of Injury model”) [28, 29]. The model consists of three sub-models: the care model, the absenteeism model, and the performance model. Costs were determined as described previously [30]. The number of patients unable to work after their accident and the duration of work absence were extracted by VeiligheidNL. In this study, the friction cost method was used, which estimates the indirect costs due to productivity loss [31]. For this outcome measure, calculations were made with data from patients aged 15 to 60 + years, in 5 year age groups of patients with one or multiple rib fractures. Cumulative duration of work absence and costs for lost productivity for different age groups were calculated by multiplying the number of patients with the mean duration of work absence per patient per age group.

### Years lived with disability (YLD)

The EQ-5D data of the questionnaire were used for calculating the number of YLD per patient. Mean and cumulative YLD were calculated for different age groups (the same as mentioned for incidence rate) in patients with either one or multiple rib fractures. To calculate the cumulative YLD, the number of patients was multiplied with the mean YLD per patient per age group.

## Results

### Incidence rate

**Fig. 1:**
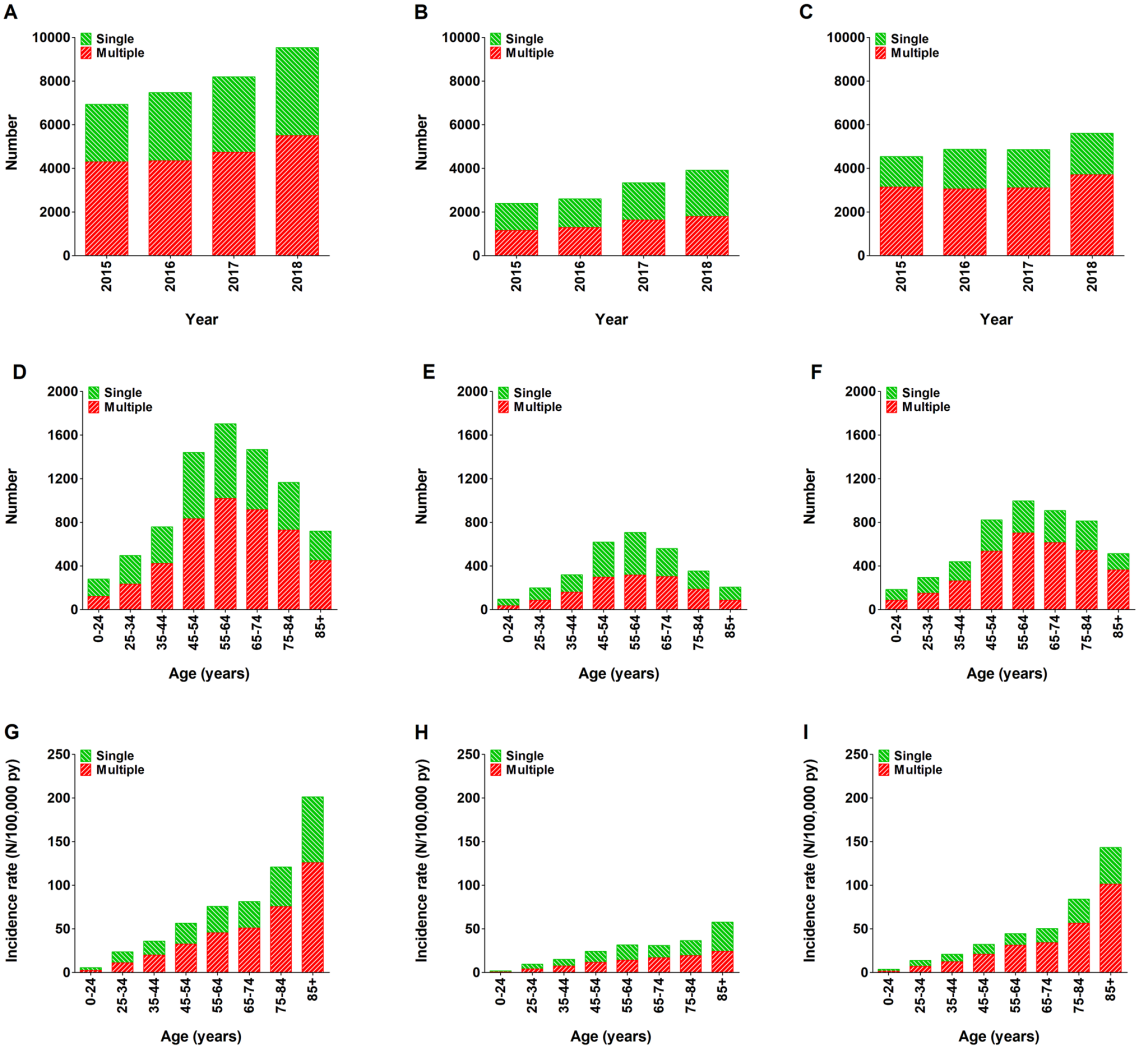
Number of patients with one or multiple (≥ 2) rib fractures per year (**a**–**c**), number of patients per age group (**d–f**), and incidence rate per age group (**g–i**). Data are presented for the total population (**a**, **d**, and **g**), as well as for non-admitted (**b**, **e**, and **h**) and admitted (**c**, **f**, and **i**) patients

During the 4-year study period, a total of 32,124 patients were registered after sustaining one or multiple traumatic rib fractures of which 19,885 (61.9%) patients required hospitalization. In total, 18,887 (58.8%) patients sustained multiple rib fractures. In the elderly (≥ 65 years or older), 72.5% of patients with multiple rib fractures required hospitalization. The total number of patients registered with rib fractures over this time period increased with 37.4% (Fig. 1a–c). The incidence rate for the total cohort was 47.1 per 100,000 person years (19.4 in patients with one rib fracture and 27.7 in patients with multiple rib fractures). The incidence rate of all admitted patients with rib fractures was 29.2 per 100,000 person years. In patients aged ≥ 65 years, the incidence rate was 107.4 per 100,000 person years (40.3 in patients with one rib fracture and 67.1 in patients with multiple rib fractures). The incidence rate of admitted patients aged ≥ 65 years with rib fractures was 71.5 per 100,000 person years. Both the total number of patients as well as the incidence rate increased strongly with age in the admitted patients and remained relatively similar in the non-admitted patients (Fig. 1d–i).

### HLOS

**Fig. 2:**
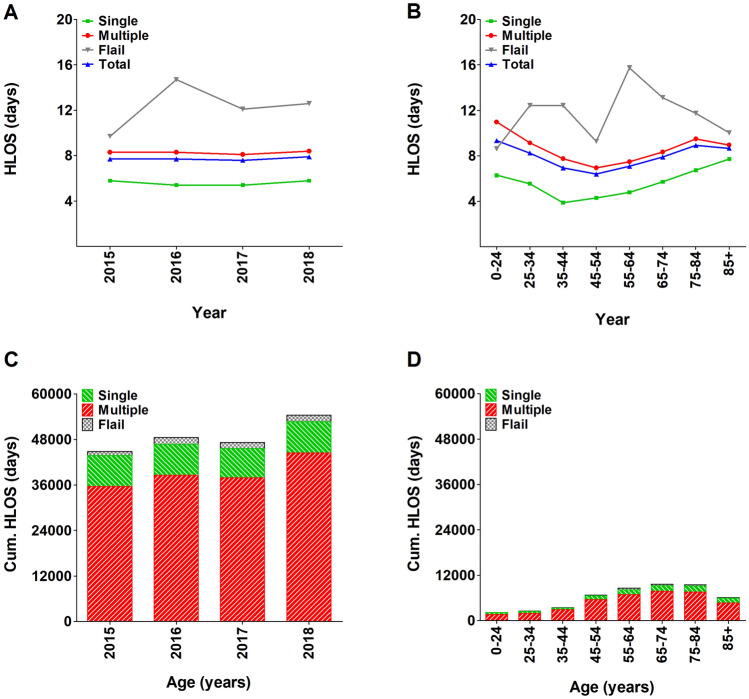
Mean HLOS in patients with one or multiple (≥ 2) rib fractures, or a flail chest per year (**a**), per age group (**b**), and cumulative HLOS per year (**c**), and per age group (**d**)

Annually, the cumulative HLOS of patients with rib fractures comprised 48,737 days. Patients with multiple rib fractures or a flail chest accounted for 83.4% of the yearly cumulative HLOS (39,117 days). Patients aged ≥ 65 years accounted for 51.8% of the average cumulative HLOS (25,256 days) in the study period. The mean HLOS per patient with rib fractures was 7.7 days: 5.6 days for patients with one rib fracture, 8.3 days for patients with multiple rib fractures, and 12.3 days for patients with a flail chest (Fig. 2a). For patients with one or multiple rib fractures, the mean HLOS decreased up to the age of 45 (3.9 days) and 55 (6.9 days), respectively, after which it increased (Fig. 2b). The age-specific distribution of the cumulative HLOS showed an increase after the age of 45 years (Fig. 2c–d).

### Costs for health care use

Nationwide, the cumulative direct health care costs for patients with rib fractures were €54.5 million per year. Admitted patients accounted for 90.5% of the cumulative direct health care costs (€49.3 million). The cumulative direct health care costs were €19.2 million for patients with one rib fracture and €35.3 million for patients with multiple rib fractures. Patients aged ≥ 65 years accounted for 59.5% (€32.4 million) of the cumulative direct health care costs.

**Fig. 3:**
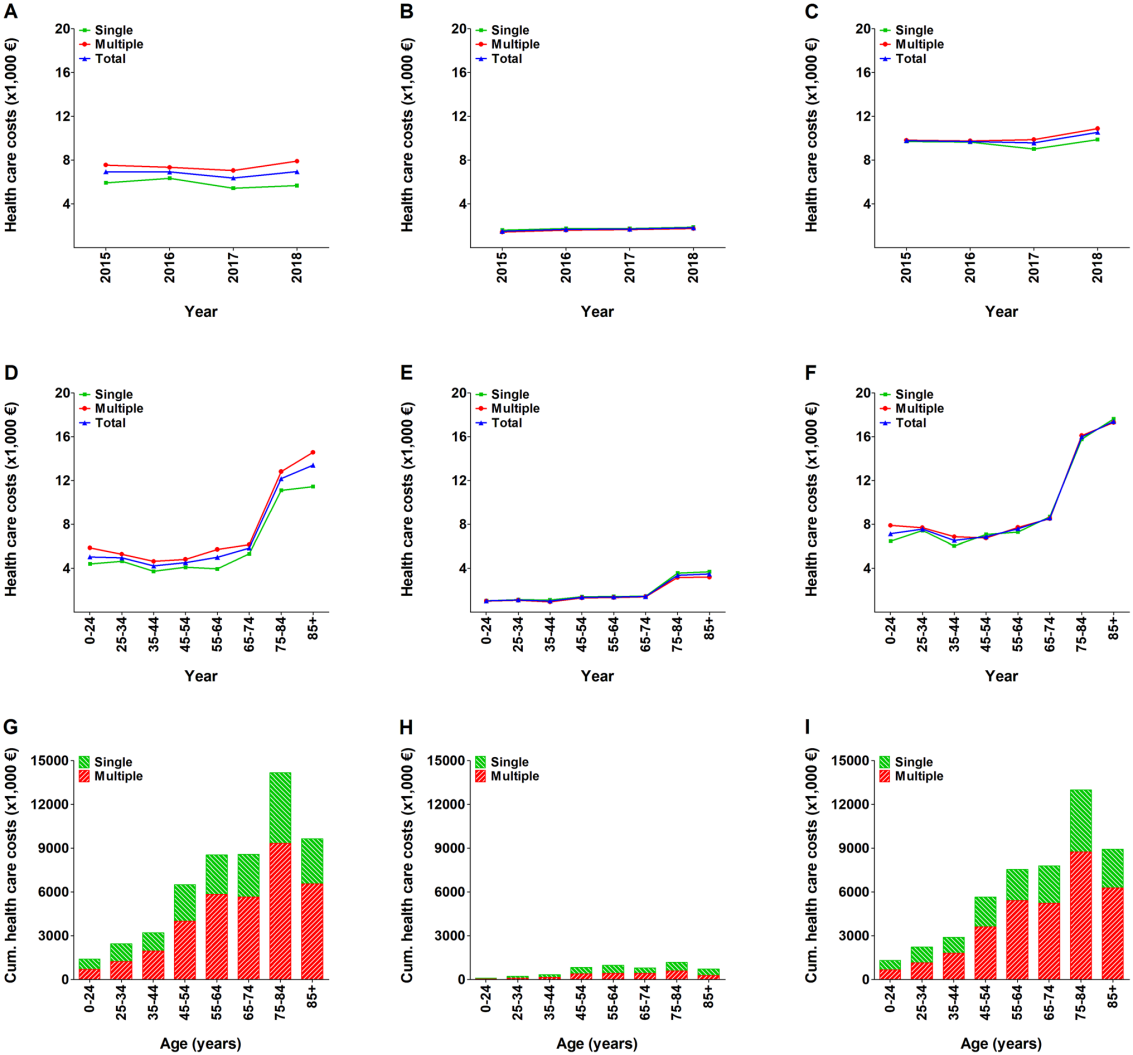
Mean direct health care costs of patients with one or multiple (≥ 2) rib fractures per year (**a**–**c**), per age group (**d**–**f**), and cumulative direct health costs per age group (**g**–**i**). Data are presented for the total population (**a**, **d**, and **g**), as well as for non-admitted (**b**, **e**, and **h**) and admitted (**c**, **f**, and **i**) patients

The mean direct health care costs for a patient with rib fractures were €6785 and were similar for patients with one or multiple rib fractures (Fig. 3a–c). The mean costs for an admitted patient with one rib fracture were €9557 and €10,115 for a patient with multiple rib fractures (Table 1). Both admitted and non-admitted patients showed an increase of costs after the age of 75 years (ranging from €4220 to €5850 up to the age of 74 years and €13,390 for patients aged 75 years and older; Fig. 3d–f). The age-dependent distribution of cumulative direct health care costs showed a sharp increase after the age of 45 years with the larger part of costs in admitted patients (Fig. 3g–i). Admitted patients older than 45 years patients covered 90.0% of the cumulative costs (€42.9 million).

**Table 1:** Direct medical costs and indirect costs for lost productivity by type of rib fracture (2015–2018)

	Total			One rib fracture			Multiple (≥ 2) rib fractures		
	*N*	Mean (€)	Cumulative (million €)	*N*	Mean (€)	Cumulative (million €)	*N*	Mean (€)	Cumulative (million €)
Health care costs (€)									
Admitted	4971	9922	49.3	1719	9557	16.4	3252	10,115	32.9
Non-admitted	3060	1689	51.7	1590	1755	2.8	1470	1619	2.4
Total	8031	6785	54.5	3309	5808	19.2	4722	7471	35.3
Costs for lost productivity (€)									
Admitted	2015	22,886	46.1	751	22,303	16.7	1265	23,233	29.4
Non-admitted	1168	2880	33.6	627	2956	1.9	541	2793	1.5
Total	3183	15,547	49.5	1378	13,496	18.6	1805	17,112	30.9
Combined direct and indirect costs (€)									
Admitted	6986	32,808	95.4	2470	31,860	33.1	4517	33,348	62.3
Non-admitted	4228	4569	85.3	2217	4711	4.7	2011	4412	3.9
Total	11,214	37,377	180.7	4687	36,571	37.8	6528	37,760	66.2

*N* number of patients

Direct and indirect costs are presented as the mean costs per patient and the cumulative costs of the entire study period, 2015–2018

### Duration of work absence and costs for lost productivity

**Fig. 4:**
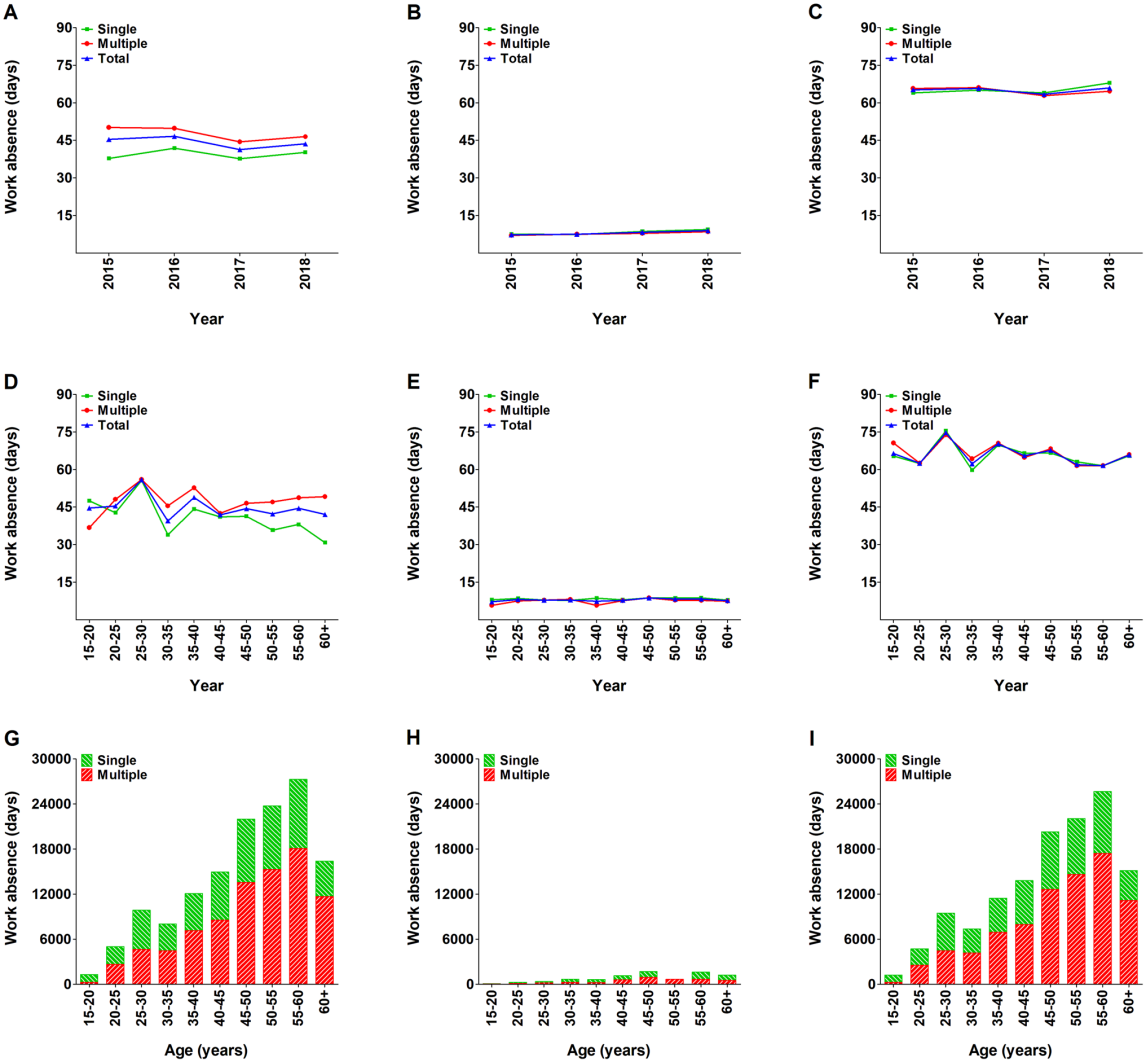
Mean duration of work absence in patients with one or multiple (≥ 2) rib fractures per year (**a**–**c**), per age group (**d**–**f**), and cumulative duration of work absence per age group (**g**–**i**). Data are presented for the total population (**a**, **d**, and **g**), as well as for non-admitted (**b**, **e**, and **h**) and admitted (**c**, **f**, and **i**) patients

In total, patients with rib fractures accounted for an annual cumulative duration of work absenteeism of 140,638 days. The mean duration of work absence per patient with rib fractures was 44.2 days (65.1 days for admitted patients and 8.1 days for non-admitted patients). The mean duration of work absence in patients with multiple rib fractures was 64.9 days for admitted patients and 7.8 days for non-admitted patients. The mean duration of work absence was stable over the years and appeared unrelated to number of rib fractures but related to hospital admission (Fig. 4a–c). In the age-specific distribution, the duration of work absence increased in the admitted patients aged 20–30 years, with patients aged 25–30 having the longest mean duration of work absence (74.7 days). After that age and in the age-specific distribution in non-admitted patients, the duration of work absence was relatively similar (Fig. 4d–f). In total, 93.3% of the cumulative duration of work absenteeism was attributable to admitted patients (131,184 days) and 61.4% to patients with multiple rib fractures (86,307 days; Fig. 4g–i).

**Fig. 5:**
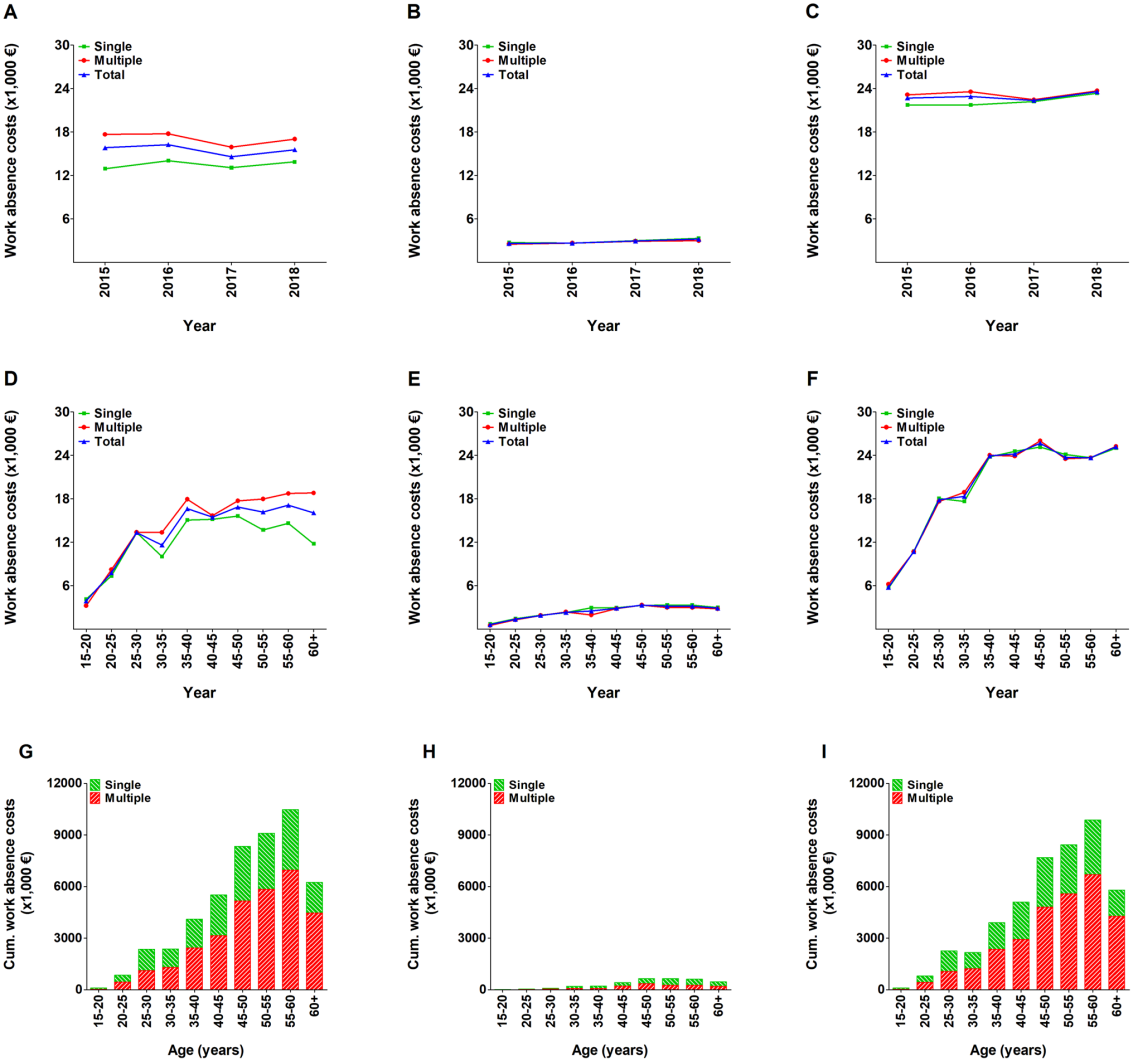
Mean indirect costs for lost productivity in patients with one or multiple (≥ 2) rib fractures per year (**a**–**c**), per age group (**d**–**f**), and cumulative costs for lost productivity per age group (**g**–**i**). Data are presented for the total population (**a**, **d**, and **g**), as well as for non-admitted (**b**, **e**, and **h**) and admitted (**c**, **f**, and **i**) patients

Annually, the cumulative costs for lost productivity were €49.5 million and the highest in patients aged 55–60 years (€10.5 million). The mean costs for lost productivity were €15,547 per patient (Table 1; Fig. 5a–c). The mean costs for lost productivity per patient increased with age for admitted patients and were highest in patients aged 55–60 years (€17,570). In both admitted and non-admitted patients, costs for lost productivity were similar for patients with one or multiple rib fractures (Fig. 5d–i). Admitted patients accounted for 93.2% of the cumulative costs and the costs increased with age (€46.1 million; Fig. 5g–i).

### Years lived with disability

**Fig. 6:**
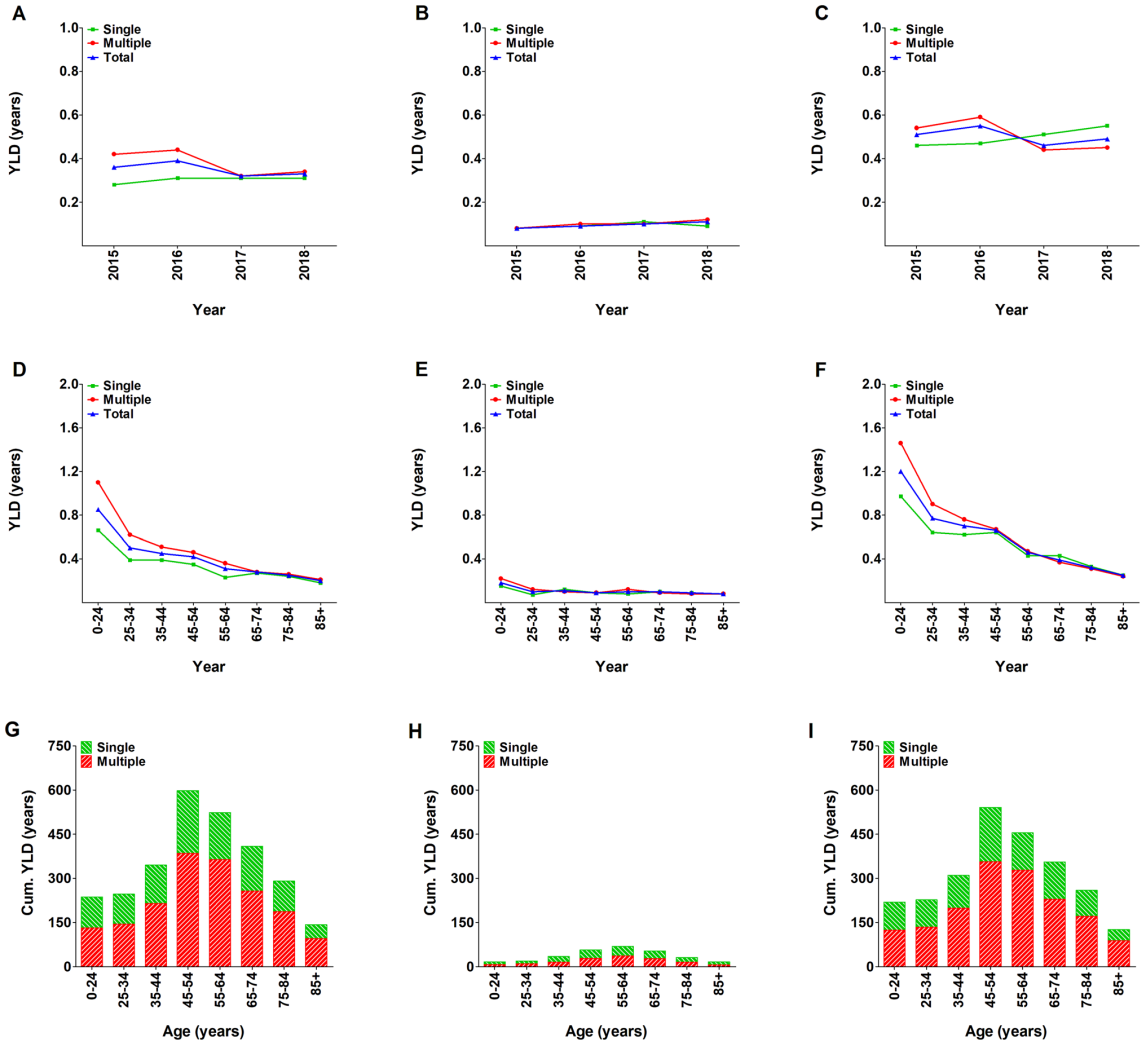
Mean years lived with disability (YLD) in patients with one or multiple (≥ 2) rib fractures per year (**a**–**c**), per age group (**d**–**f**), and cumulative YLD per age group (**g**–**i**). Data are presented for the total population (**a**, **d**, and **g**), as well as for non-admitted (**b**, **e**, and **h**) and admitted (**c**, **f**, and **i**) patients

The average cumulative YLD for patients with rib fractures were 2792 years per year with a mean YLD per patient of 0.35 years. The mean YLD per patient was 0.5 years in admitted patients and 0.1 years in non-admitted patients (Fig. 6a–c). In the age-specific distribution, the YLD decreased with age (highest in patients aged 0–24, 0.85 years) and was most pronounced in admitted patients (Fig. 6d–f). The average YLD for the working population age groups was 0.39 years and 0.25 years for patients aged ≥ 65 years. Patients in the working population age groups (25–64 years) had a cumulative YLD of 1713 years which was 61.4% of the total YLD (Fig. 6g–i).

## Discussion

This epidemiologic nationwide study is the first to describe health care use and costs in admitted and non-admitted patients with rib fractures. Patients registered with rib fractures were more prevalent over time and the incidence rate increased with age. Almost two-thirds of patients with rib fractures required hospital admission with a mean stay of about 8 days with associated significant direct and indirect costs, and over 2 months of work absenteeism. The direct and indirect costs, duration of work absence, and years lived with disability remained stable during the study period and appeared unrelated to having sustained one or multiple (≥ 2) rib fractures but increased considerably with age and in admitted patients compared with non-admitted patients.

The incidence rate of 29 per 100,000 person years for admitted patients is similar to the incidence rate demonstrated by a recent study focusing only on admitted patients [18]. The incidence rate for the total cohort of 47 per 100,000 person years showed that rib fractures are common and not all patients require admission. To put things in perspective, the total incidence rate of patients with a humeral fracture is 40 per 100,000 person years [32]. The incidence rate increased sharply with age, from 34 to 107 per 100,000 person years in patients aged ≤ 65 years and ≥ 65 years, respectively. The incidence rate of 72 per 100,000 person years for admitted elderly patients is again in line with previous literature [18, 20]. Furthermore, the increase of 37% of the total number of patients with rib fractures between 2015 and 2018 demonstrates that rib fractures are a common and increasing problem, especially among the elderly [33]. Age is a known risk factor for mortality in patients with rib fractures [5, 9, 10, 12]. This highlights the need for improvement of preventive measures and a possibly more aggressive multimodal therapy in this type of patient.

While the cumulative HLOS of 48,737 days is not as high as for, *e.g*., patients with foot and ankle injuries (58,708 days), the mean HLOS of almost 8 days is higher than for patients with a humeral or tibia shaft fracture in the Netherlands [24, 32, 34]. The mean HLOS increased with severity of the injury (single or multiple rib fractures, or a flail chest), but without data on concomitant injuries, need for additional interventions (*e.g.,* chest tube, surgical stabilization of rib fractures (SSRF), or video-assisted thoracoscopic surgery (VATS), or complications such as pneumonia, the true impact on the HLOS attributable to sustaining rib fractures, is not known. Nonetheless, it has been shown that almost half of the patients with rib fractures are polytraumatized (*i.e.,* ISS > 15) patients and require Intensive Care Unit admission, demonstrating that rib fractures are a marker of severe injury [35].

The annual cumulative direct health costs were €54.5 million of which 90% was accounted for by admitted patients. Similar age-dependent mean HLOS for patients with one or multiple rib fractures suggests that the high direct health care costs might mostly be accounted for by out of hospital care. The age-dependent distribution of direct health costs showed a sharp increase after the age of 45 years. Thus, efforts to improve the preventive measures and both in and out of hospital care should not only focus on the elderly but possibly on patients as young as 45 years [11]. Again, as there was no insight into treatment parameters such as need for medicinal, radiologic, or operative interventions, the exact impact of rib fracture injuries on health care costs could not be distilled.

With a mean duration of work absence of 44 days and cumulative indirect costs for lost productivity of €49.5 million, rib fractures pose a serious societal health burden. While many possible confounding variables are not known for this outcome, these results are reinforced by previous studies indicating long-term disability and long work absenteeism after rib fractures [15–17, 36]. The mean direct health care costs for a patient with rib fractures were €6785 which is higher than that of patients with ankle or foot injuries (€3461), but lower than for patients with a humeral fracture or a hip fracture (€8864 and €10,458, respectively) [32, 34, 37]. Comparing these mean costs per patient with previous literature is difficult as most studies have focused solely on in-hospital costs of patients with a flail chest, injury severity score (ISS) of ≥ 16 and compared operatively and nonoperatively treated patients [22, 38–40]. With no information in this study on the received treatment modality, ISS, ICU admission, or costs in patients with flail chest, paralleling these results is not feasible. To our knowledge, the duration of work absenteeism and consequent costs for lost productivity in patients with rib fractures have not been studied before. However, rates of 33% to 42% of patients have been reported to be unable to work at their pre-injury capacity at three and even 12 months post-injury, respectively [41, 42]. These associated high costs with lengthy HLOS and work absenteeism, which increase with age, might consequently indicate the need for a different approach to the patient with rib fractures. Currently, most patients with rib fractures are treated nonoperatively, and SSRF is intended for the younger patient with a high ISS [42, 43]. Based on the age-dependent increase in HLOS and costs in admitted patients, the benefit of restoring the chest wall biomechanics through SSRF might actually be higher in the older population in which some physiological decompensation is present [44].

While this epidemiologic study is the first to evaluate health care use and costs in patients with rib fractures in the Netherlands, interpretation of the results should be done in the light of several limitations. First, miscoding and incomplete data are inherent to using nationwide or large registries such as the DISS and HDR. As rib fractures are often accompanied by other severe injuries, rib fractures might not have been registered, resulting in an underestimation of the incidence rate of rib fractures. Currently, different national registries are used for registration of trauma data and consequent research. The DISS database only records data from Dutch emergency departments, the HDR and the Dutch National Trauma Registry from admitted patients. The different registries provide complementary useful data, but currently cannot be linked. Thus, to provide adequate and complete data on for example the incidence rate, health care use, and costs, one national registry which includes complete short- and long-term data on both admitted and non-admitted patients is urgently needed.

Second, patients from the DISS were registered as having sustained one or multiple rib fractures where multiple was defined as 2 or more rib fractures. In current literature, multiple rib fractures are defined as having three or more rib fractures, regardless of side or adjacency [42, 45, 46]. Only for the HLOS, which was covered by the HDR, a third subgroup, patients with a flail chest, was available. This discrepancy in defining subgroups of patients with rib fractures hinders generalizability and providing practicable conclusions. In addition, rib fractures were diagnosed by either chest CT or radiograph, or both. The use of chest CT is more sensitive in the delineation of rib fractures as it finds two to three additional rib fractures compared with radiograph [47, 48]. Therefore, patients who were registered as having sustained one rib fracture, diagnosed through chest radiograph, might have been registered as a patient with multiple rib fractures if a chest CT had been made. In this light, this might have introduced selection bias in diagnosing a patient with or without rib fractures, but also in the number of rib fractures. The diagnosis of these additional rib fractures on chest CT might result in increased admission rates and subsequently increased total health care costs, but the effect of the diagnostic modality on in-hospital outcome such as complication rate and mortality remains unclear [47, 49, 50]. This limitation does, however, reflect daily practice as a large number of hospitals and EDs still use chest radiograph as the primary diagnostic modality in patients with rib fractures.

Third, the distinction between patients with rib fractures as primary diagnosis or secondary diagnosis was not possible with the available data. Thus, patients were admitted with rib fractures and not because of rib fractures. This distinction in rib fracture as primary or secondary diagnosis could have helped in providing insight in the individual impact of rib fractures on the outcome measures. Another covariate which was not available, was treatment modality. Outcomes such as costs for health care, lost productivity, and YLD might be influenced by whether a patient underwent SSRF or nonoperative treatment. To date, studies have shown significant in-hospital differences in health care costs between treatment modalities in patients with rib fractures, but data on the effect of treatment modality on outcomes such as lost productivity or YLD remains limited [22, 39, 40]. Also, comorbidity, concomitant injury characteristics, trauma mechanism, the ISS, and abbreviated injury scale scores were not known for these patients. Therefore, the outcome measures could not be corrected for possible confounding variables. This might explain the relatively similar direct and indirect costs as well as years lived with disability in patients with either one or multiple rib fractures with admitted patients contributing most to these outcomes. While this stresses the need for improved nationwide registries, these results show that sustaining even a single rib fracture is a marker of severe injury as it might be accompanied by a long duration of health care use, work absence, and high costs.

In conclusion, this epidemiologic study shows that the number of patients registered with rib fractures has been increasing over time and the incidence rate increases with age. Although it was not possible with the current available data to prove causality between outcomes and rib fractures specifically, it does demonstrate that sustaining rib fractures indicates severe injury and may be associated with lengthy HLOS and work absenteeism as well as high direct and indirect costs. The duration of work absence and associated costs increase with age and are considerably higher in admitted patients than in non-admitted patients. The outcomes appear to be similar in patients with one or multiple rib fractures. However, due to the non-standard definitions of rib fractures, the lack of additional individual patient data, and impossibility to combine data of the different national registries, the generalizability and results of this study should be interpreted with caution.

## Data Availability

The data used to support the findings of this study are restricted by the Dutch Injury Surveillance System (DISS), maintained by VeiligheidNL, and the Hospital Discharge Registry (HDR). Requests for access to these data should be made to VeiligheidNL and the HDR.
